# Impact of Social Media Use on Segmentation of Dining out Behavior Among Younger Generations: A Case Study in South Korea

**DOI:** 10.3390/foods13244146

**Published:** 2024-12-21

**Authors:** Jin A Jang, Ji-Myung Kim, Hyosun Jung

**Affiliations:** 1 Department of Wellness Food Therapy, Ansan University, 155, Ansandaehak-ro, Sangnok-gu, Ansan-si 15328, Gyeonggi-do, Republic of Korea; jjang3913@ansan.ac.kr; 2 Department of Food and Nutritional Science, Shinhan University, 95, Hoam-ro, Uijeongbu 11644, Gyeonggi-do, Republic of Korea; 3 Center for Converging Humanities, Kyung Hee University, 26 Kyungheedae-ro, Dongdaemun-gu, Seoul 02447, Republic of Korea; chefcook@khu.ac.kr

**Keywords:** social network service, variety-seeking tendency, recommended information utilization, eating out, young generations

## Abstract

This study examined how eating out behavior and variety-seeking tendency in food choice (VARSEEK) differ depending on social network service (SNS) use and recommended information utilization (SURU), focusing on Korean generation Z youth. To this end, participants were categorized as high, middle, or low based on their SURU score; eating out behavior, as well as VARSEEK, were then compared across the three groups. The results indicated that higher SURU scores were associated with a higher frequency of cooking, a higher frequency of eating out, a higher average cost of eating out per person, and a greater tendency to perceive oneself as gourmet. In relation to VARSEEK, the high and middle SURU score groups demonstrated significantly higher mean scores than the low group. This finding suggests that the greater the SURU level, the greater the food neophilic inclination, expressing an affinity for unique, unfamiliar, or exotic cuisine and a willingness to experiment with novel recipes. Consequently, SURU leads to more frequent eating out, resulting in consumers expanding into a food neophilic tendency to try more diverse and new foods. Based on these results, SURU can be a useful indicator for segmenting food- and restaurant-related markets; consumers with a high level of SURU are a group to pay attention to in marketing as they can be tested when introducing new foods into the market.

## 1. Introduction

As of October 2023, 5.3 billion people around the world use the internet, accounting for 65.7% of the world’s population. Of these, 4.95 billion, or 61.4% of the world’s population, are social media users [1]. Media and technologies that facilitate social interaction (i.e., social media) are favored by young adults (20–30 years old), who spend more time on media and technology each day than on any other activity [2,3,4]. Among the various content shared, sharing food content offers a way for individuals to express their identity, emotions, culture, and memories [5]. Posting photos of food on social network services (SNSs) and choosing a restaurant to visit through SNS has become not only an SNS trend but also a dining trend [6]. The number of users using SNS to find delicious meals is rapidly increasing, and consumers explore restaurants to ensure quality, enjoy popular tastes, and reduce selection risk [7].

According to one report, respondents between the ages of 18 and 35 search for food content on Instagram about five days a week, and 30% of respondents are reluctant to visit restaurants with a low Instagram presence [8]. Following the COVID-19 pandemic (2021–2022), the frequency of eating out and spending on dining out among Texas residents increased [9]. Similarly, in Korea, takeout orders increased by 35.0%, delivery by 34.4%, cooking by 26.4%, and home meal replacement (HMR) food by 22.6% [10]. When looking at the eating patterns of Korean college students, 32.1% of them use home-cooked meals and 69.9% of them eat out (including delivery food, convenience food, and eating out), which means that eating out rather than making home-cooked meals is the main type of eating [11]; even at home, they prefer meal kits rather than cooking themselves [12]. At this point of social and cultural transformation, SNS use and recommended information utilization when purchasing food, eating out can act as a medium to increase consumers’ eating out behavior.

As social media technology develops, consumers receive more information for food-related purposes, such as descriptions of dishes or recipes, comparisons of foods, and evaluations of restaurants, which ultimately leads to decisions such as selection, purchase, and eating [13]. When consumers come across various food photos and recipes through SNSs, they feel the desire to try new foods [14]. Young people’s food choices have also expanded through recipe information readily available through social media; in addition, they can demonstrate their food preparation skills and express themselves in the kitchen by posting photos and posts of their own food [15]. According to Oh et al. [16], the more consumers enjoy using SNSs related to eating out and find the information useful, the greater their motivation to eat out for enjoyment and sociality. Furthermore, the more people use SNSs for dining-related information, the more their positive expectations about dining out increase. Furthermore, as recommended information utilization (SURU) level increases, consumers visit restaurants at a higher rate. They visit restaurants, share food photos and experiences on SNSs, build relationships with people, search for restaurants they want, try new foods, and share those experiences again on SNSs [17]. In addition, it was reported that SNS activities that share and recommend various types of content and information increase the intention to visit restaurants and purchase intention [18], and the motivation to eat out and satisfaction increase through SNS use [16]. In addition, as social media strongly intervenes in the lifestyles and daily behaviors of young people, it can serve as a unique opportunity to change the modern approach to supporting healthy eating habits [19]. Social media platforms are considered reliable information resources, and 52% of young people use social media to seek advice on health issues [20]. As the influence of SNSs on the food and dining sector grows, related research is being conducted, but research on the characteristics of the groups that use SNSs is still insufficient.

Variety-seeking tendency (VARSEEK) is a personal characteristic variable of consumers that refers to the pursuit of something unique or new among consumers [21]. According to previous research on VARSEEK, consumers often pursue a significant amount of variety when choosing more than one option from a set of alternatives, even when they are allowed to repeatedly select their favorites [22,23]. According to a study by Kim and Kim [24], although restaurant consumers with a strong tendency to seek variety perceived a high level of customization benefits, its link with the intention to revisit was low. In addition, consumers with a tendency to pursue diversity are more likely to actively accept and trust social commerce; the tendency to pursue diversity has also been shown to have a positive effect on social commerce [25]. The VARSEEK scale [26] is a common tool used to assess food neophilia. Developed in the context of consumer research, VARSEEK has been primarily validated as a tool to assess food novelty as a consumer characteristic in order to develop marketing strategies for new foods [27]. Previous studies on food neophilia have been conducted mainly from the perspective of consumers’ attitudes toward the tendency to seek diversity in new foods, such as the food diversity of and neophobia towards edible insects [28], unfamiliar foods from different cultures [29], and less traditional ingredients in product innovations [30]. Studies have shown that individuals with higher scores on the food neophobia scale (FNS) report lower levels of openness [31,32] and extraversion [32]. However, little is known about the relationship between SURU and the tendency to seek variety from the perspective of food neophilia.

Based on the results of existing research, SURU is thought to be related to eating out behavior as well as to the tendency to seek variety, but few studies have examined this relationship, especially among young adults. Therefore, this study aims to comprehensively examine the relationship between Korean young people’s eating out behavior and VARSEEK according to SURU. To further explore the relationship between VARSEEK and SURU in relation to FNS, we also examined how the FNS-related measure “Motivation to Eat New Foods scale (MENF)” affects SURU.

## 2. Materials and Methods

### 2.1. Participants and Data Collection

This study targeted consumers in their 20s and 30s who currently have SNS accounts and live in the Seoul-Gyeonggi area, in the center of Korea. These age groups in South Korea have similar latent profiles in terms of social media usage behaviors and have been reported to be more active and engaged in social media than other age groups [30,31]. Participants were selected using a convenience sampling method through an online site for two days from 8 June to 9 June 2023. The questionnaire was administered only to those who gave their consent after receiving a letter explaining the purpose and methodology of this study. They responded to a structured questionnaire through an online survey. A total of 300 surveys were collected, and 294 (98.0%) responses were used for the final analysis after excluding 6 surveys that included unfaithful responses through the data cleaning process. Participant recruitment and online surveys were conducted through Embrain, Inc. (Seoul, Republic of Korea). To ensure ethical rigor, a research protocol describing the purpose, content, and methods of this study was submitted to the Shinhan University Institutional Review Board (SHIRB-202305-HR-185-01) for review and approval.

### 2.2. Survey Tools

In order to analyze consumers’ eating out behavior and food variety-seeking tendency according to SNS use and recommendation information in this study, some questions were selected from previous studies and reorganized to fit this study. First, as a measure of consumers’ use of SNS and utilization of recommendation information, four questions asked how much participants use SNS in general and how much they rely on SNS recommendations when eating out, cooking, and buying food for each topic using a 5-point Likert scale (1 = never, 5 = always) [17]. We then examined consumers’ SNS usage behavior by asking them to indicate on a nominal scale their interests in SNSs, the platforms they use, the average time they spend on SNS per day, the frequency of posting posts and photos on SNSs, the platforms they use when searching for restaurant recommendations, and the restaurants they search for [33,34,35]. To determine consumers’ eating out behavior, the frequency of cooking at home, the frequency of dining out, and the average cost of dining out by individual were investigated using a nominal scale [36,37,38]. They were also asked if they considered themselves foodies based on a 5-point Likert scale. Meanwhile, the VARSEEK scale developed by Van Trijp and Steenkamp [26] was used to determine consumers’ food neophilia tendencies toward food. The scale of the original English text was converted to the Korean scale through a translation and back-translation process by three food experts fluent in both Korean and English. VARSEEK consists of 8 questions, including 1 reverse question: 7 questions about food neophilic traits, such as whether the individual is curious and willing to try the most unusual menu items, new recipes, unfamiliar foods, and exotic foods, and 1 question about food neophobia, such as whether the individual prefers to eat familiar foods. All questions were measured using a 7-point scale (1 = not at all, 7 = very much). Considering consistency with other variables, it was recoded on a 5-point scale and used in this study. To further explore the relationship between SURU and VARSEEK in relation to FNS, we examined the FNS-related scale and the MENF together. The original FNS was based on the assumption that food neophobia and neophilia are simply two opposite poles of the same continuum between avoiding and approaching unfamiliar foods [26]. Recently, however, this assumption has been increasingly challenged by researchers [31], who point out that food neophobia and neophilia are closely related but conceptually distinct constructs. In light of this, Nezlek et al. [39] developed the MENF, a scale to analyze the separate two concepts of FNS, such as approach and avoidance motivation in new foods. The present study used it to examine how the two perspectives on motivation to eat new foods relate to SURU. This study included a total of 10 items, with 5 items focused on approach (such as trying new foods and enjoying learning about them) and 5 items focused on avoidance (such as not trusting new foods and fearing trying them). The translation process of the English scales and the use of the measurement scales were the same as in VARSEEK. To determine the demographic characteristics of all study subjects, questions were asked about gender, age, marital status, household composition, occupation, education level, and household monthly income, all of which were measured on a nominal scale.

### 2.3. Data Analysis

All statistical analyses in this study used IBM Series (version 21.0, Chicago, IL, USA). A correlation analysis of the variables used in the SURU scale produced a Pearson’s correlation coefficient. To categorize the subjects according to the degree of SURU and to evaluate its reliability and validity, an exploratory factor analysis with principal component analysis was conducted, and the reliability (Cronbach’s α) of the extracted factors was analyzed. K-means clustering analysis was performed on the factor scores derived from the factorization, and the results were classified into three clusters. The demographic characteristics of the three SURU groups were analyzed using frequency analysis to identify overall trends, and differences between groups were analyzed through chi-square testing. To examine the differences in the mean scores of the SURU questions in each group, an analysis of covariance was performed, adjusting for the age of the subjects, and the results were compared using Duncan’s multiple comparison test. A chi-square test was conducted to analyze the frequency differences in SNS usage behavior and eating out behavior among the three SURU groups. Among them, cooking frequency, dining-out frequency, and average monthly personal food expenses were tested with analysis of variance (ANOVA); differences among groups were analyzed and compared using Duncan’s multiple comparison test. The same analysis was conducted for self-perceived foodies and VARSEEK according to SURU. In addition, the effect of VARSEEK and MENF on SURU was analyzed through regression analysis.

## 3. Results

### 3.1. Exploratory Factor Analysis, Reliability Analysis, and Clustering Based on SURU

An exploratory factor analysis was conducted to assess the reliability and validity of the scales used in this study. All four questions met the criteria for factor extraction: factor loadings of 0.4 or higher and eigenvalues of 1 or higher. As a result of the factor analysis, a total of one factor was extracted, which was named SURU considering the characteristics of the constituent questions. Therefore, SURU can be defined as the degree of use of SNSs and recommendation information from SNSs when purchasing food and eating out. The Kaiser–Meyer–Olkin (KMO) value was 0.795, indicating a good fit for factor analysis, and Bartlett’s test was significant (*p* < 0.001) at 541.805, resulting in a total explanatory power of 68.012%. The reliability analysis of the factors showed a Cronbach’s α value of 0.835. A K-means cluster analysis was performed, with the factor scores derived through a factor analysis and classified into three clusters; the mean score for each question of SURU was compared across groups (Table 1). As a result, there was a significant difference in the SURU average score among the three groups at *p* < 0.001. Using this information, each group was classified as high, medium, or low according to the average score ranking of the questions. In all variables, each group showed significant differences in mean scores at *p* < 0.001. In particular, the three groups showed distinct differences in the utilization of SNS recommendation information when cooking/eating out/purchasing food. However, in terms of SNS use, there was no significant difference between the two groups, except for the low group, which had the lowest average score (*p* < 0.001). Thus, the high group is the most likely to use SNSs and utilize SNS recommendations for eating out and purchasing food, whereas the low group is the opposite. The middle group has a high level of SNS use but a somewhat lower level of utilization of SNS recommendations when eating out and purchasing food; it can be interpreted as a group with a lower level of interest in food than the high group.

### 3.2. Demographics of SURU Groups

The demographic characteristics of all study participants and the SURU groups are shown in Table 2. The total number of participants was 294, with a slightly higher percentage of female participants than male participants. In terms of age, participants in their 20s and 30s were equally represented; in terms of marital status, single people were overwhelmingly represented. In terms of occupation, the most common positions were office worker and student. The majority of participants had graduated college, and the participants earned more than KRW 6 million as their average monthly income, followed by less than KRW 2 to 3 million. Among the SURU groups, no significant differences emerged between the groups, except for age and occupation. In terms of age, participants in the high group were mostly in their 20s, whereas in the low group, those in their 30s were more common. The middle group had no significant difference between the two age groups, although 54.7% were in their 30s. Meanwhile, in terms of jobs, office workers were most common in the high group. Students were the most prevalent in the middle group, and service workers and unemployed individuals were more prevalent in the low group than in the other two groups.

### 3.3. SNS Usage Behavior Based on SURU

The results of analyzing SNS usage behavior by SURU group are shown in Table 3. Significant differences emerged among the three groups in terms of interests on SNSs, average time spent on SNSs per day, frequency of posting on SNSs, and restaurants that respondents mainly searched for. When it comes to social media interests, dining out scored the highest among all participants. Among the three groups, food and dining out were highest in the high group (48.7%), followed by the middle group (37.3%) and low group (16.9%). The high and middle groups showed similar interests in food and dining out, followed by entertainment and fashion, but the low group differed, with entertainment being the highest, followed by fashion and sports/exercise. Regarding the average time spent on SNSs per day, the most frequent response in the high and middle groups was less than 2 to 5 h, but in the low group, the most frequent response was less than 1 to 2 h. The percentages of respondents that recorded a frequency of more than 5 h in the high, middle, and low groups were 12.9%, 11.6%, and 1.2%, respectively. The rates of this response in the high and middle groups were similar, but the low group showed a significantly lower rate. Regarding the frequency of posting on SNSs, the high group most frequently reported doing so once every few weeks and several times a week. The middle group most commonly reported a rate of once every few months and several times a week, while the low group most frequently responded with never and once every few months. The frequency rates of giving the response of 1 to 2 or more times a day were 26.6%, 16.3%, and 3.6%, respectively, with the high group showing the highest frequency. When it comes to mainly searched restaurants, restaurants near the participants’ current location were selected significantly higher than other responses in all groups. Other characteristics of each group are that the high group had the highest percentage of searching for trendy restaurants, while the low group had the highest rate of responding that they rarely searched for restaurants.

### 3.4. Eating out Behavior Based on SURU

Table 4 presents the results of comparing participants’ eating out behavior based on SURU. The results of the chi-square test among the three groups’ frequency of cooking at home showed a significant difference at the *p* < 0.01 level. In particular, the three groups were most likely to cook at home more than 2–3 times a week (63.7%, 68.6%, and 46.5% for the high, middle, and low groups, respectively). Converting the frequencies within these questions into a seven-point scale and comparing them showed that the scores for the high and middle groups were significantly higher than that of the low group, and there was no significant difference between the high and middle groups. Regarding the frequency of eating out, the chi-square test did not show a significant difference among the three groups, but when the data were scaled to seven points and compared, this frequency was found to be significantly higher in the high and middle groups. As for the result of the chi-square test for the average cost of eating out per person, the most common response in the high, middle, and low groups was a cost exceeding KRW 300,000, accounting for 39.6%, 31.4%, and 20.2%, respectively, of the responses in each group; when the data were analyzed by ANOVA, the high group scored significantly higher than the other groups. In the question about whether the participants consider themselves foodies, a significant difference emerged among the three groups, with the high group having the highest score, followed by the middle and low groups.

### 3.5. VARSEEK According to SURU

The ANOVA of VARSEEK for the SURU group (Table 5) found that the high and middle groups had significantly higher average scores than the low group at the *p* < 0.01 level. An examination by question demonstrated significant differences for seven of the eight questions, with the reverse question being the only one not to show such differences. Among these questions, the high group scored the highest on two questions: the most unusual items and food eaten by people from other countries. For the remaining questions, the high and middle groups scored the highest, while the low group scored the lowest. Among all the questions in all groups, the highest average score was found for “I am eager to know what kinds of foods people from other countries eat”. This result indicates that younger consumers in their 20s and 30s are generally interested in foreign and unfamiliar foods and have a strong neophilic tendency to try them.

The correlations between SURU, VARSEEK, and MENF were analyzed (Table 6), and all correlations were significant, except for the relationship between SURU and positive MENF (motivation to avoid new foods). Among them, SURU, VARSEEK, and positive MENF all showed significant positive correlations, but negative MENF (motivation to avoid new foods) and other variables showed significant negative correlations.

The results of the regression analysis of the effects of VARSEEK and MENF on SURU are shown in Table 7. VARSEEK and the motivation to approach new foods each had a significant positive effect on SURU. On the other hand, motivation to avoid new foods showed no significant effect on SURU.

## 4. Discussion

This study examined how eating out behaviors and food variety-seeking tendencies differ according to SURU, focusing on millennials and Generation Z. To this end, a survey was conducted and analyzed on 294 consumers in their 20s and 30s living in the Seoul-Gyeonggi area of Korea who currently have SNS accounts.

The summary and discussion of the results of this study are as follows: A factor analysis considering participants’ degree of SURU was conducted to extract one common factor, and it was divided into three groups according to this degree. Through this, it was confirmed that the high group are consumers who use SNSs frequently and actively utilize SNS recommendation information when cooking, eating out, and purchasing food. In addition, the middle group has a high usage of SNSs but a low usage of SNS recommendations when cooking, eating out, and buying food. The low group has a low usage of both.

The analysis of SNS usage behavior by SURU group showed significant differences among the three groups in terms of their interests on SNSs, average time spent on SNSs per day, frequency of posting on SNSs, and the restaurants they mainly search for. Among them, the proportion of food and eating out among SNS interests, the average time spent on SNSs per day, and the frequency of posting on SNSs were the highest for the high group, and trendy restaurants scored the highest among the restaurants this group searched for on SNSs, indicating that members of this group are sensitive to trends when eating out. In general, no significant differences were found among the groups in terms of favorite social media platforms or favorite platforms for searching for food and dining out recommendations, but the ranking of social media platforms differed in each case. Korean consumers use Instagram the most in general, but when it comes to searching for recommendations for food and dining out, they use the local platform Naver Blog the most. Participants also mentioned YouTube as one of their favorite platforms for general use, as well as for searching for information in the food and dining space. Meanwhile, globally, Facebook is the most popular, followed by YouTube [40]. Lewis [41] noted the growing role of both laypeople and experts, such as professional chefs, in providing expert advice on food and cooking in the digital food landscape; such growth is most evident on YouTube, where food videos and channels account for a significant proportion of content. In addition, Lewis et al. [42] reported on digital media use in Australian households, with housewives accessing recipes online and watching YouTube clips in the kitchen to learn how to cook. Meanwhile, Yost et al. [43], who examined the impact of social media on the performance of food and beverage companies, found that Instagram had a greater impact than Twitter and Facebook and reported that the posts with the most engagement among related content were related to taste. Instagram is an image-based social media platform primarily used for self-promotion and social networking with friends; a categorization of a sample of Instagram images showed that the food category accounted for more than 10% of the posted images, ranking after the selfie, friends, and activity categories [44].

When we analyzed the relationship between SNSs and the cooking and eating behavior of the three groups, we found that the higher the SURU score, the higher the frequency of eating out, the higher the average cost of dining out per person, and the more likely they were to perceive themselves as foodies. These results suggest that the higher the SURU, the higher the interest in eating out and the higher the gastronomic preferences. The recent global spread of middle-class lifestyles and the resulting phenomena of consumerism, identity-related labor, and self-branding have led to a proliferation of foodie-centric social media. Social media is also becoming more customized and personalized when it comes to an individual’s lifestyle and culture; food is a popular and influential topic. Foodie-centric social media is taking shape through online platforms, offering advice on food and cooking [45]. In response, the role of everyday people with expertise is growing, and the lines between professional, celebrity, and amateur chefs are becoming increasingly blurred. Dining reviews posted by these key opinion leaders—including photos of the food and atmosphere and ratings and comments on food quality and price—have been found to have a significant and positive impact on young generations’ intentions to visit restaurants [46]. Younger generations are also turning to social media for ideas, inspiration, and tips, not just on cooking techniques, but at all stages of the culinary journey: deciding what to make, learning how to prepare it, and actually cooking or baking it [47]. As a result, the number of views of food and recipe content continues to grow [48,49]. In this context, it is not surprising that the high-SURU group in this study showed high tendencies for eating behaviors and gastronomic perceptions. In other words, by indirectly experiencing others’ experiences through SNSs, individuals maximize and expand the range of their own experiences. It has also been reported that experiencing food through words or images on social media can increase cravings for that food [50], and the results of this study can be understood in the same way. According to the grounded cognition theory of desire, food cues (e.g., words and images) can trigger a rewarding simulation or re-experiencing of food consumption, which can lead to cravings for particularly attractive foods [51]. In fact, it has been reported that seeing food words or images activates the gustatory and reward regions of the brain, triggering a re-experience or simulation of the food, just like when you taste it yourself [52]. Therefore, it is believed that the more often consumers encounter food and dining-related content on SNSs, the more interested and curious they become about food, which leads to a better diet and to dining out, which in turn becomes content on SNSs; the process thus becomes a cycle.

In terms of VARSEEK based on the SURU group, the high and middle groups showed significantly higher mean scores than the low group. In addition, the correlation between SURU and VARSEEK question scores showed a significant positive correlation with all questions except the reverse question. These results suggest that the higher the SURU level, the more food neophilic an individual is, showing the characteristics of liking and being curious about unusual, unfamiliar, or exotic foods and liking to try new recipes. These results also tie into earlier findings about eating out behavior and self-perceived foodie. In other words, SURU reinforces eating out behavior, which may lead to a greater propensity for food diversity and a willingness to try new foods. In addition, self-identifying as a foodie can also be linked to a propensity to try and enjoy different foods. Whether people consume a variety of foods depends on, among other things, their willingness to try new and unfamiliar foods [53], and food neophilia describes an apparent willingness to try these [54]. VARSEEK [26] is a common instrument to assess food neophilia [31,32]. Previous studies have reported higher levels of openness and extraversion among individuals with higher levels of food neophilia, as well as higher social class, income, education, and younger age [53]. It has also been reported that higher food neophilia tendencies are associated with higher levels of sensation-seeking and greater variation in food consumption [26]; a higher willingness to try unfamiliar foods is understood to be connected to a higher openness to new experiences [54]. Given that VARSEEK has a significant positive effect on SURU in this study, it is likely that the aforementioned food neophilia tendencies are reflected in SURU users. In other words, they are more open and extroverted, pursue higher sensations, and want to enjoy new experiences through a diverse diet. Furthermore, it is likely that the abundance of food-related words and images on social media contributed to this experience. Previous studies [27,55] have reported higher levels of food neophilia among people in the upper class than those in the working or middle classes, with the explanation being that people in the upper class are more likely to be exposed to unfamiliar foods through international travel and greater knowledge of other cultures and cuisines. However, recently, with the development of SNSs, it is possible to easily encounter the cultures and foods of other countries, as well as new food ingredients, recipes, and food-related trends, through various forms of global content. This shift is driven by technology, as consumers can be clustered into various interest groups based on interactions such as food reviews, forums, blogs, and feedback on social media; algorithms such as recommendation systems provide information that is most relevant to their interests [56]. Therefore, with the development of technology and the accumulation of vast amounts of data, the use of SNSs is expected to contribute significantly to increasing consumers’ propensity to seek food diversity. On the other hand, VARSEEK scores have been reported to be negatively correlated with food neophobia, generalized neophobia, and trait anxiety [27]. However, the results of the present study indicate a different trend, with no significant differences between the SURU groups on the food neophobia items within the VARSEEK scale and a negative correlation—but not a significant difference—with SURU. In addition, the results of analyzing the effect of motivation to avoid new foods on SURU also showed that it was not significant. In other words, SURU is not used for food neophobia, and low SURU does not indicate high food neophobia.

This study has several academic and practical implications. First, this study holds significance in academia as it uncovers the traits of SNS users pertaining to eating out which have previously not been researched in detail. South Korea is a global digital powerhouse, and the results of our study will be even more meaningful in that they will allow people to experience and utilize a variety of dining out cultures through SNSs. In particular, we found that SURU leads directly to cooking frequency and dining out consumption, which may extend to food neophilia, where consumers are more willing to try different and new foods. It also reaffirms the importance of social media to young consumers’ eating out behaviors. In addition, it is meaningful to expand consumer understanding by applying SURU in relation to food neophilia characteristics. Especially for younger consumers, social media has become integral in their lives, opening up new ideas and experiences. It is considered a vital stepping stone in their exploration of new foods. This study offers practical insights into the social media usage and the cooking and eating out behavior of millennial and Generation Z consumers, which will prove useful for food outlets as consumer marketing information. Given that SURU relates to cooking frequency, eating out, and openness to new foods, we propose that SURU can serve as an innovative indicator for segmenting the eating out market. Consumers exhibiting high SURU levels are a viable demographic for testing new foods during market introduction. Marketing efforts should also target this group for optimal results.

This study also has certain limitations. First, because the study participants were limited to millennials and Generation Z living in a metropolitan area in South Korea, it is difficult to generalize the results of this study to the entire Korean population. Second, since the sample was selected using convenience sampling, there is a possibility that there may be problems with the representativeness of some samples. Therefore, it is believed that longitudinal studies that conduct surveys at different time points will be necessary in future studies. Third, there is a shortage of prior research on the characteristics of SNS users in general, not just those related to restaurants, which limits the in-depth interpretation of the results of this study. Fourth, this study dealt with integrated and general SNS use related to eating out, but no analysis was conducted on detailed areas related to them. In future research, further analysis should be conducted in precise areas related to food and dining out, including SNS channels and the extent of active SNS use (e.g., periodic data uploads vs. solely viewing). Diverse research can also reveal consumer characteristics, such as comparisons of SURU-related gastronomic tendencies using gastronomy-related scales.

## 5. Conclusions

This study sought to understand the characteristics of SNS users related to eating out, which had previously been insufficiently studied, through SNS usage behavior, cooking and eating out behavior, and the tendency to pursue food diversity. SURU led to eating out more often and, consequently, consumers developed a food neophilic tendency to try more diverse and new foods. This finding suggests that SURU can be used as a useful indicator in segmenting food and restaurant-related markets, and consumers with a high level of SURU are a particularly important group that can be tested when introducing new foods into the market, warranting more attention in marketing. The results of this study are expected to provide important insights into consumer behavior research and SNS marketing plans related to SNS use in the food service context.

## Figures and Tables

**Table 1:** Characteristics of SURU groups.

Variables	High Group (*N* = 124)	Middle Group (*N* = 86)	Low Group (*N* = 84)	F-Value
Degree of SNS use	4.35 ± 0.90 ^b(1)^	4.41 ± 0.68 ^b^	2.64 ± 1.12 ^a^	107.84 ***^(2)^
Utilizing SNS-recommended information when dining out	4.27 ± 4.99 ^c^	2.83 ± 0.80 ^b^	1.98 ± 0.58 ^a^	361.69 ***
Utilizing SNS-recommended information when cooking	4.15 ± 0.68 ^c^	2.92 ± 0.80 ^b^	1.90 ± 0.80 ^a^	227.13 ***
Utilizing SNS-recommended information when purchasing food	3.99 ± 0.55 ^c^	2.78 ± 0.76 ^b^	1.82 ± 0.71 ^a^	275.75 ***
Average score	4.17 ± 0.04 ^c^	3.24 ± 0.04 ^b^	2.09 ± 0.05 ^a^	690.40 ***

(1) Means with different letters (a~c) within the same row are significantly different by Duncan’s multiple range test (mean ± SD). (2) *** *p* < 0.001.

**Table 2:** Demographics of respondents.

Variables		Total	High Group (*N* = 124)	Middle Group (*N* = 86)	Low Group (*N* = 84)	χ^2^
Gender	Male	145 (49.3) ^(1)^	56 (45.2)	42 (54.8)	47 (56.0)	2.34
	Female	149 (50.7)	68 (48.8)	44 (51.2)	37 (44.0)	
Age	20s	147 (50.0)	78 (62.9)	39 (45.3)	30 (35.7)	15.86 ***^(2)^
	30s	147 (50.0)	46 (37.1)	47 (54.7)	54 (64.3)	
Marital status	Single	229 (77.9)	100 (80.6)	66 (76.7)	63 (75.0)	1.02
	Married	65 (22.1)	24 (19.4)	20 (23.3)	21 (25.0)	
Occupation	Student	61 (20.7)	25 (20.2)	21 (24.4)	15 (17.9)	25.58 *
	Office worker	135 (45.9)	65 (52.4)	36 (41.9)	34 (40.5)	
	Housewife	11 (3.7)	6 (4.8)	2 (2.3)	3 (3.6)	
	Business	9 (3.1)	3 (2.4)	3 (3.5)	3 (3.6)	
	Professional	22 (7.5)	12 (9.7)	8 (9.3)	2 (2.4)	
	Service	27 (9.2)	6 (4.8)	8 (9.3)	13 (15.5)	
	None	20 (6.8)	6 (4.8)	3 (3.5)	11 (13.1)	
	Other	9 (3.1)	1 (0.8)	5 (5.8)	3 (3.6)	
Education	High school	31 (10.5)	10 (8.1)	9 (10.5)	12 (14.3)	5.11
	In college	43 (14.6)	17 (13.7)	17 (19.8)	9 (10.7)	
	College	199 (67.7)	88 (71.0)	55 (64.0)	56 (66.7)	
	Graduate school	21 (7.1)	9 (7.3)	5 (5.8)	7 (8.3)	
Average monthly income (KRW)	Less than 2 million	19 (6.5)	3 (2.4)	5 (5.8)	11 (13.1)	14.71
	Less than 2 to 3 million	62 (21.1)	23 (18.5)	20 (23.3)	19 (22.6)	
	Less than 3 to 4 million	47 (16.0)	18 (14.5)	14 (16.3)	15 (17.9)	
	Less than 4 to 5 million	44 (15.0)	18 (14.5)	15 (17.4)	11 (13.1)	
	Less than 5 to 6 million	35 (11.9)	18 (14.5)	8 (9.3)	9 (10.7)	
	More than 6 million	87 (29.6)	44 (35.5)	24 (27.9)	19 (22.6)	

(1) *N* (%). (2) * *p* < 0.05, *** *p* < 0.001.

**Table 3:** SNS usage behavior according to SURU groups.

		Total	High Group (*N* = 124)	Middle Group (*N* = 86)	Low Group (*N* = 84)	χ^2^
Interests on SNSs	Food and dining out	102 (36.6) ^(1)^	58 (48.7)	31 (37.3)	13 (16.9)	25.69 **^(2)^
	Fashion	44 (15.8)	16 (13.4)	11 (13.3)	17 (22.1)	
	Pets	20 (7.2)	7 (5.9)	8 (9.6)	5 (6.5)	
	Sports/exercise	32 (11.5)	9 (7.6)	9 (10.8)	14 (18.2)	
	Entertainment	59 (21.1)	19 (16.0)	20 (24.1)	20 (26.0)	
	Etc.	22 (5.1)	10 (8.4)	4 (4.8)	8 (10.4)	
Average time on SNSs per day	Less than 1 h	50 (17.0)	8 (6.5)	10 (11.6)	32 (38.1)	49.03 ***
	Less than 1–2 h	106 (36.1)	42 (33.9)	31 (36.0)	33 (39.3)	
	Less than 2–5 h	111 (37.8)	58 (46.8)	35 (40.7)	18 (21.4)	
	More than 5 h	27 (9.2)	16 (12.9)	10 (11.6)	1 (1.2)	
Frequency of posting on SNSs	Never	51 (17.3)	5 (4.0)	8 (9.3)	38 (45.2)	94.36 ***
	Once every few months	59 (20.1)	14 (11.3)	21 (24.4)	24 (28.6)	
	Once every few weeks	68 (23.1)	38 (30.6)	18 (20.9)	12 (14.3)	
	A few times a week	66 (22.4)	34 (27.4)	25 (29.1)	7 (8.3)	
	Several times a day	50 (17.0)	33 (26.6)	14 (16.3)	3 (3.6)	
Favorite platforms for food shopping and dining recommendations	Instagram	82 (28.5)	40 (32.3)	24 (27.9)	18 (23.1)	8.82
	Naver Blog	127 (44.1)	45 (36.3)	45 (52.3)	37 (47.4)	
	YouTube	64 (22.2)	32 (25.8)	12 (14.0)	20 (25.6)	
	Others	15 (5.2)	7 (5.6)	5 (5.8)	3 (3.8)	
Restaurants mainly searched for	Restaurants near current location	203 (69.0)	83 (66.9)	61 (70.9)	59 (70.2)	19.01 *
	Trendy restaurants	47 (16.0)	29 (23.4)	10 (11.6)	8 (9.5)	
	New restaurants	15 (5.1)	4 (3.2)	7 (8.1)	4 (4.8)	
	Rarely search	10 (3.4)	1 (0.8)	2 (2.3)	7 (8.3)	
	Others	19 (6.5)	7 (5.6)	6 (7.0)	6 (7.1)	

(1) *N* (%). (2) * *p* < 0.05, ** *p* < 0.01, *** *p* < 0.001.

**Table 4:** Eating out behavior by SURU group.

		Total	High Group (*N* = 124)	Middle Group (*N* = 86)	Low Group (*N* = 84)	χ^2^
Cooking frequency	Never	25 (8.5) ^(1)^	6 (4.8)	5 (5.8)	14 (16.7)	26.73 **^(2)^
	Once a month	18 (6.1)	5 (4.0)	4 (4.7)	9 (10.7)	
	Once every two weeks	20 (6.8)	9 (7.3)	6 (7.0)	5 (6.0)	
	Once a week	54 (18.4)	25 (20.2)	12 (14.0)	17 (20.2)	
	2–3 times per week	108 (36.7)	49 (39.5)	38 (44.2)	21 (25.0)	
	4–5 times per week	42 (14.3)	13 (10.5)	16 (18.6)	13 (15.5)	
	At least once a day	27 (9.2)	17 (13.7)	5 (5.8)	5 (6.0)	
	Average score ^(3)^	4.48 ± 1.62	4.72 ± 0.49 ^b(7)^	4.65 ± 1.45 ^b^	3.96 ± 2.85 ^a^	6.30 **
Dining out frequency	Never	12 (4.1)	2 (1.6)	4 (4.7)	6 (7.1)	18.73
	Once a month	28 (9.5)	8 (6.5)	6 (7.0)	14 (16.7)	
	Once every two weeks	54 (18.4)	20 (16.1)	16 (18.6)	18 (21.4)	
	Once a week	74 (25.2)	30 (12.2)	25 (29.1)	19 (22.6)	
	2–3 times per week	83 (28.2)	43 (34.7)	21 (24.4)	19 (22.6)	
	4–5 times per week	28 (9.5)	13 (10.5)	8 (9.3)	7 (8.3)	
	At least once a day	15 (5.1)	8 (6.5)	6 (7.0)	1 (1.2)	
	Average score ^(4)^	4.13 ± 1.43	4.41 ± 1.33 ^b^	4.17 ± 1.46 ^b^	3.67 ± 1.45 ^a^	7.14 **
Average cost of dining out per person	Less than 100 thousand	38 (12.9)	5 (4.0)	13 (15.1)	20 (23.8)	30.55 **
	Less than 100–200 thousand	80 (27.2)	27 (21.8)	26 (30.2)	27 (32.1)	
	Less than 200–300 thousand	83 (28.2)	43 (34.7)	20 (23.3)	20 (23.8)	
	Less than 300–400 thousand	49 (16.7)	27 (21.8)	16 (18.6)	6 (7.1)	
	Less than 400–500 thousand	27 (9.2)	14 (11.3)	5 (5.8)	8 (9.5)	
	500 thousand or more	17 (5.8)	8 (6.5)	6 (7.0)	3 (3.6)	
	Average score ^(5)^	2.99 ± 1.36	3.34 ± 1.24 ^b^	2.91 ± 1.40 ^a^	2.57 ± 1.37 ^a^	8.64 ***
Consider yourself a foodie		3.04 ± 0.92 ^(6)^	3.36 ± 0.91 ^c^	3.00 ± 0.83 ^b^	2.61 ± 0.84 ^a^	19.34 ***

(1) *N* (%). (2) ** *p* < 0.01, *** *p* < 0.001. (3), (4), Seven-point Likert scale (1—never; 7—at least once a day). (5) Six-point Likert scale (1—less than 100 thousand; 6—500 thousand or more). (6) Five-point Likert scale (1—not at all; 5—completely agree). (7) Means with different letters (a~c) within the same row are significantly different by Duncan’s multiple range test (Mean ± SD).

**Table 5:** Variety-seeking tendency in food choice according to SURU groups.

VARSEEK	High Group (*N* = 124)	Middle Group (*N* = 86)	Low Group (*N* = 84)	*F*-Value
When I eat out, I like to try the most unusual items, even if I am not sure I would like them.	3.18 ± 1.14 ^(1)b(2)^	2.98 ± 1.14 ^a^	2.77 ± 1.21 ^a^	3.02 *
While preparing foods or snacks, I like to try out new recipes.	3.27 ± 1.06 ^b^	3.20 ± 0.05 ^b^	2.73 ± 1.13 ^a^	7.01 **
I think it is fun to try out food items one is not familiar with.	3.67 ± 0.91 ^b^	3.42 ± 1.09 ^b^	3.11 ± 1.15 ^a^	7.34 **
I am eager to know what kind of foods people from other countries eat.	3.81 ± 0.89 ^b^	3.57 ± 0.87 ^ab^	3.39 ± 1.10 ^a^	5.34 **
I like to eat exotic foods.	3.56 ± 0.94 ^b^	3.49 ± 0.87 ^b^	3.18 ± 1.01 ^a^	3.99 *
Items on the menu that I am unfamiliar with make me curious.	3.49 ± 1.01 ^b^	3.43 ± 0.95 ^b^	3.04 ± 1.13 ^a^	5.30 **
I prefer to eat food products I am used to. (R)	2.20 ± 0.77	2.24 ± 0.66	2.31 ± 0.90	0.47
I am curious about food products I am not familiar with.	3.35 ± 0.93 ^b^	3.45 ± 0.91 ^b^	3.06 ± 0.98 ^a^	4.05 *
Average score	3.31 ± 0.67 ^b^	3.22 ± 0.75 ^b^	2.94 ± 0.86 ^a^	6.16 **

(1) Five-point Likert scale (1—not at all; 5—completely agree). (2) Means with different letters (a,b) within the same row are significantly different by Duncan’s multiple range test (Mean ± SD). * *p* < 0.05, ** *p* < 0.01.

**Table 6:** Correlation analysis between SURU, VARSEEK, and MENF.

	SURU	VARSEEK	Motivation to Approach New Foods	Motivation to Avoid New Foods
SURU	1			
VARSEEK	0.223 **^(1)^	1		
Positive MENF	0.280 **	0.867 **	1	
Negative MENF	−0.019	−0.253 **	−0.223 **	1

(1) ** *p* < 0.01.

**Table 7:** The effect of VARSEEK and MENF on SURU.

	Standardized Coefficients	t-Value	*p*-Value
Constant		7.221	0.000 ***
VARSEEK	0.140	1.361	0.048 *
Positive MENF (motivation to approach new foods)	0.255	2.283	0.023 *
Negative MENF (motivation to avoid new foods)	−0.045	−0.775	0.439

F-value = 8.473 ***, R^2^ = 0.284 ***, * *p* < 0.05, *** *p* < 0.001.

## Data Availability

The original contributions presented in the study are included in the article, further inquiries can be directed to the corresponding author.
